# A Rare Case of Hemorrhagic Giant Adrenal Myelolipoma: Radiographic and Pathologic Correlation

**DOI:** 10.7759/cureus.17353

**Published:** 2021-08-21

**Authors:** Yanni S Zulia, Dheeraj Gopireddy, Sindhu Kumar, Anastasia Singareddy, Chandana Lall

**Affiliations:** 1 Radiology, Lake Erie College of Osteopathic Medicine, Bradenton, USA; 2 Radiology, University of Florida College of Medicine, Jacksonville, USA; 3 Radiology, University of North Florida, Jacksonville, USA; 4 Abdominal Imaging, University of Florida College of Medicine, Jacksonville, USA

**Keywords:** myelolipomas, spontaneous hemorrhage, adrenal glands, computer tomography scan, body mri, t1 fat saturated sequence, t2 weighted sequence, laparoscopic resection

## Abstract

Myelolipomas are rare benign tumors made up of adipose and hematopoietic tissue that commonly occur in the adrenal glands unilaterally. Spontaneous hemorrhage occurs in < 5% of these tumors, and often present as large masses. A 50-year-old male presented with right flank pain that had been growing increasingly worse over a two-week period. Contrast-enhanced Computed Tomography (CT) revealed a large suprarenal 15-cm mass exerting mass effect on the kidney and liver along with possible hemorrhage. T1 fat saturated and T2 non-fat saturated magnetic resonance imaging (MRI) confirmed the diagnosis of a myelolipoma with hemorrhage. The patient was treated with surgical resection of the mass and the follow-up pathology report confirmed a giant hemorrhagic adrenal myelolipoma. Spontaneous hemorrhage of a large myelolipoma measuring 15 cm is a rare entity and the correct imaging needs to be done in order to carry out the appropriate treatment.

## Introduction

A myelolipoma is a rare benign tumor consisting of lipomatous and hematopoietic tissue [1]. These tumors usually present in the adrenal glands and are often unilateral [1]. Rarely, they do appear at other sites of the human body and have been reported in the mediastinum, spleen, kidney, bones, thorax, and nasal cavity [2]. Myelolipomas are usually detected incidentally on imaging modalities including Ultrasound (US), CT, and MRI [1]. With widespread use of these imaging modalities, incidental detection is becoming more prevalent. Adrenal myelolipomas are the second most common primary adrenal incidentalomas, representing 6-16% of adrenal incidentalomas [2]. True incidence is unknown due to the high percentage of asymptomatic cases, but it is thought to be 0.08%-0.4% [3].

Adrenal myelolipomas are diagnosed around the age of 51 on average, with no difference reported between male and female [1]. These tumors are often smaller than 4 cm in diameter, with the largest being reported at 31 x 24.5 x 11.5 cm^3^. Myelolipomas are usually asymptomatic, however patients may feel pain if they are large or have hemorrhage [1]. Hemorrhagic myelolipomas are a rare entity with spontaneous tumor ruptures occurring in only 4.5% of tumors [4]. These bleeds can cause retroperitoneal, intraperitoneal, or intralesional bleeding and most tend to occur in lesions greater than 10 cm [5,6]. We describe a case of a hemorrhagic adrenal myelolipoma in a 50-year-old male presenting with progressive right flank plain and discuss the literature.

## Case presentation

**Figure 1:**
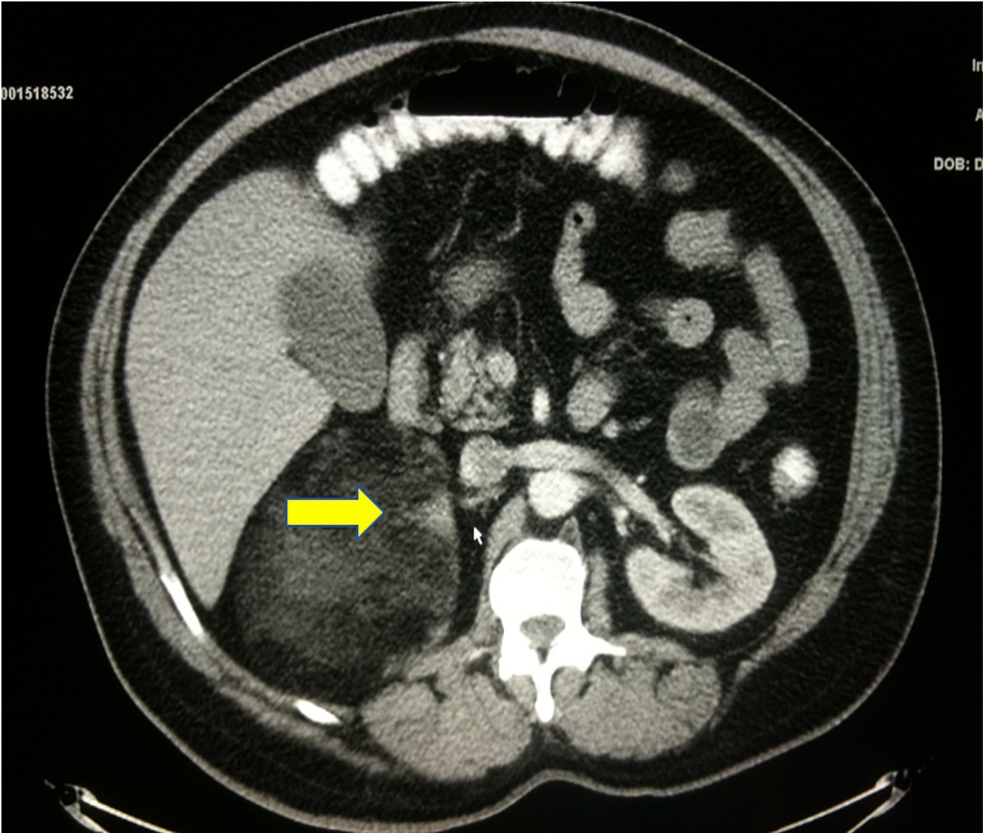
Contrast Enhanced CT Arterial Phase - Axial View Image demonstrates a large suprarenal, predominantly fatty mass with areas of soft tissue attenuation and focal hemorrhage (yellow arrow).

**Figure 2:**
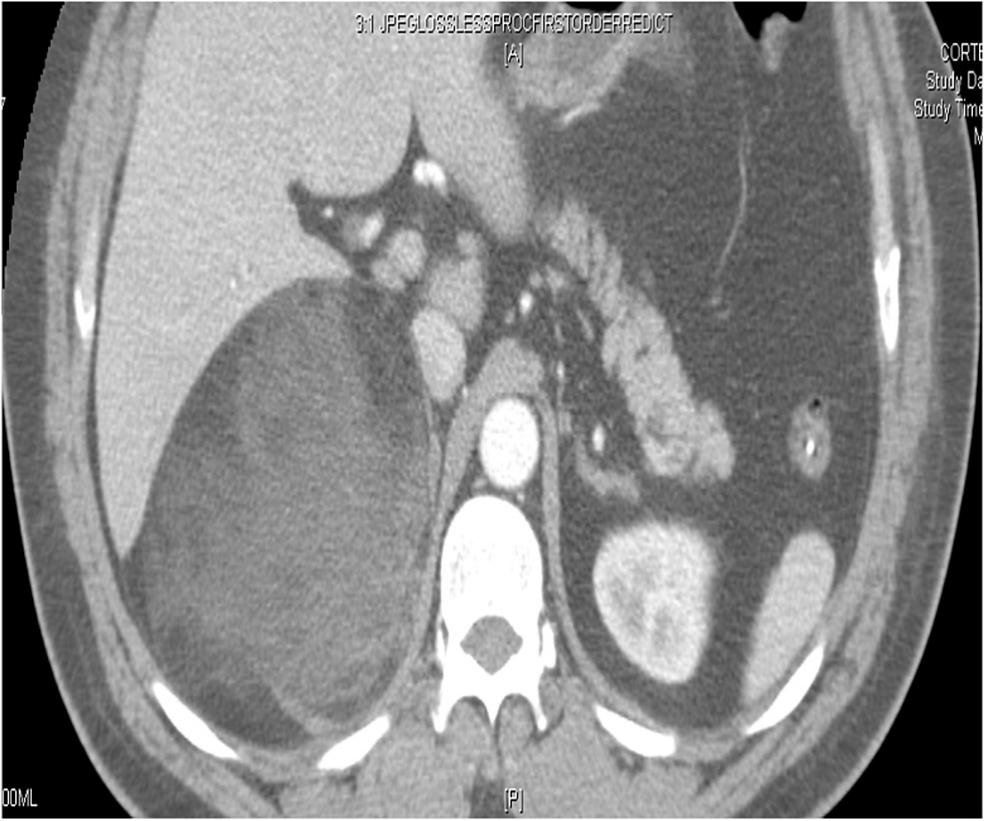
CT Arterial Phase - Axial View Image demonstrates a 15 cm retroperitoneal mass exerting mass effect on the kidney and liver.

**Figure 3:**
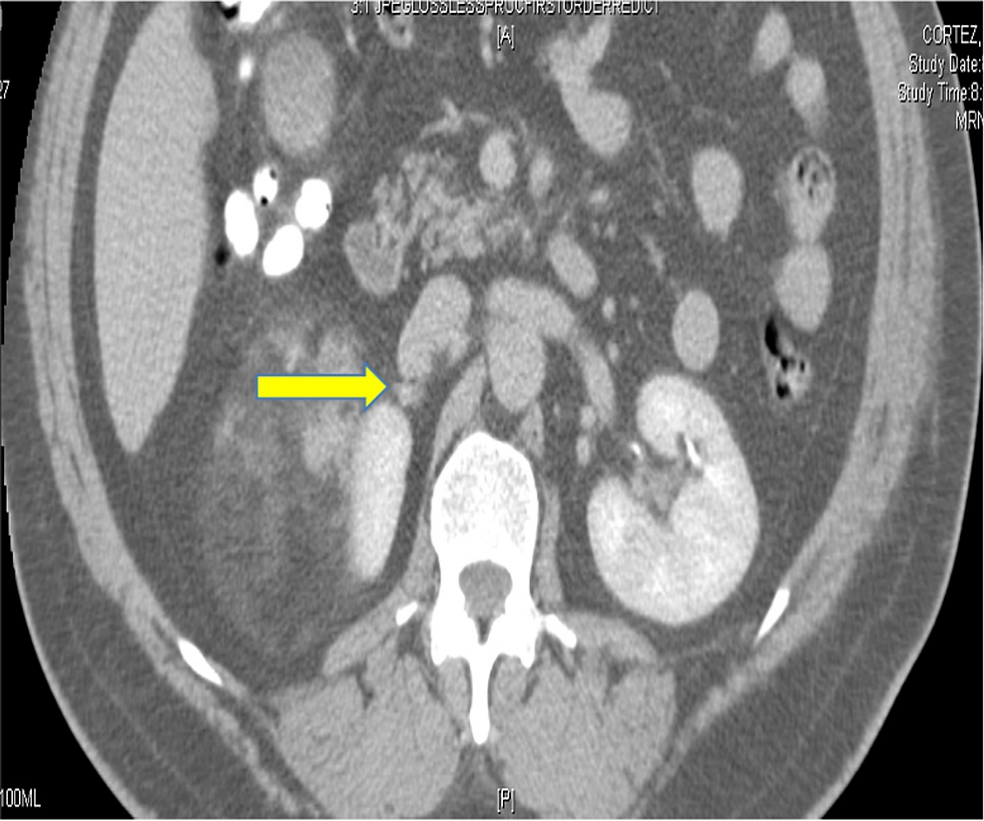
CT Portal Venous Phase - Axial View Image demonstrates a draining right adrenal vein (yellow arrow) which was ligated during surgery.

**Figure 4:**
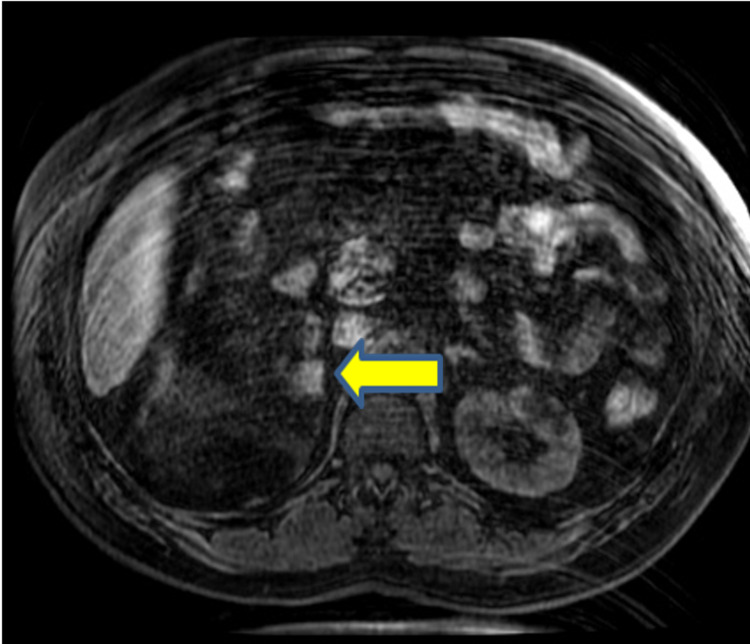
MRI Pre T1 Fat Saturated - Axial View Image shows foci of high signal indicating hemorrhage (yellow arrow).

**Figure 5:**
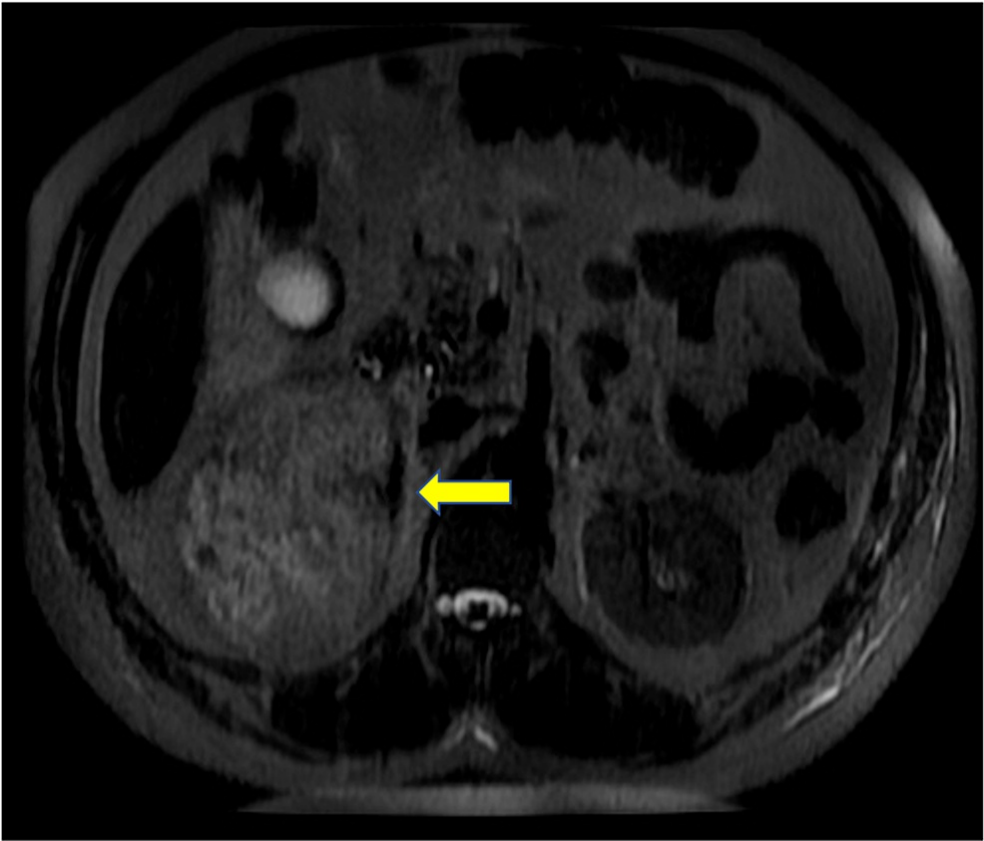
MRI T2 Single Shot Fast Spin Echo - Axial View Image shows areas of focal hemorrhage with blooming (yellow arrow).

**Figure 6:**
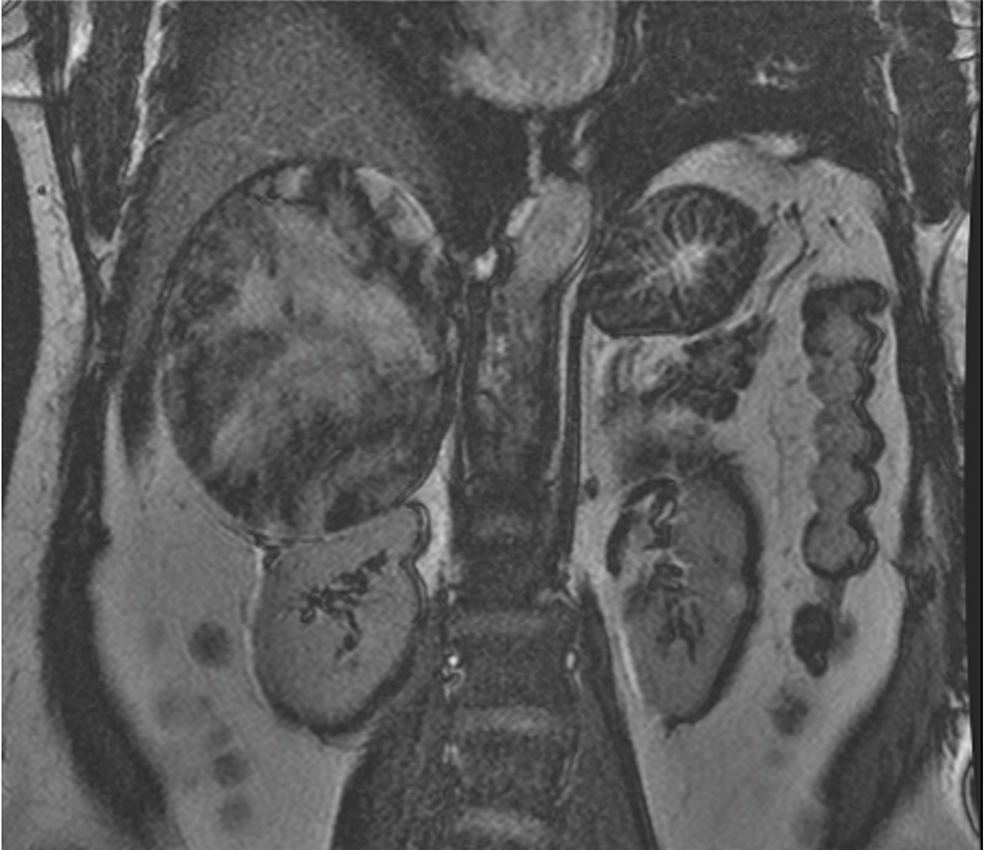
MRI 2-d Fast Imaging Employing Steady-State Acquisition Non-Fat Saturated - Coronal View Image demonstrates a large right adrenal gland lesion with mass effect on right kidney.

**Figure 7:**
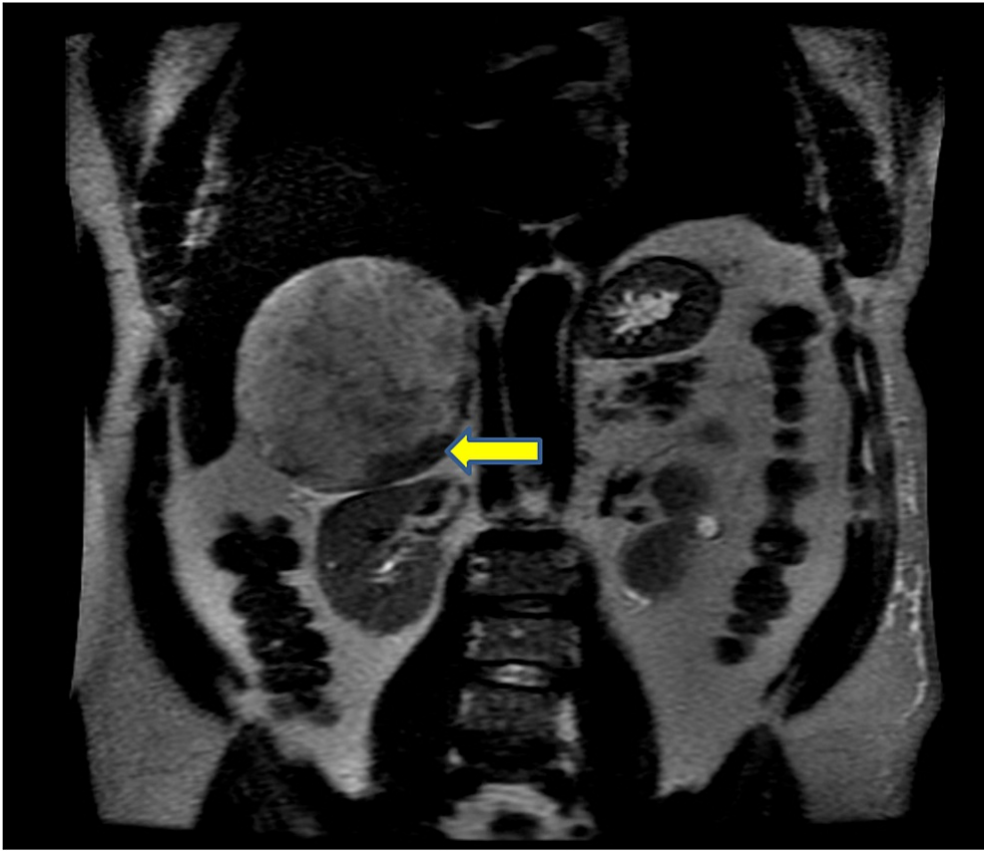
MRI T2 Non-Fat Saturated - Coronal View Image shows hemorrhage in the inferior aspect of the mass (yellow arrow).

**Figure 8:**
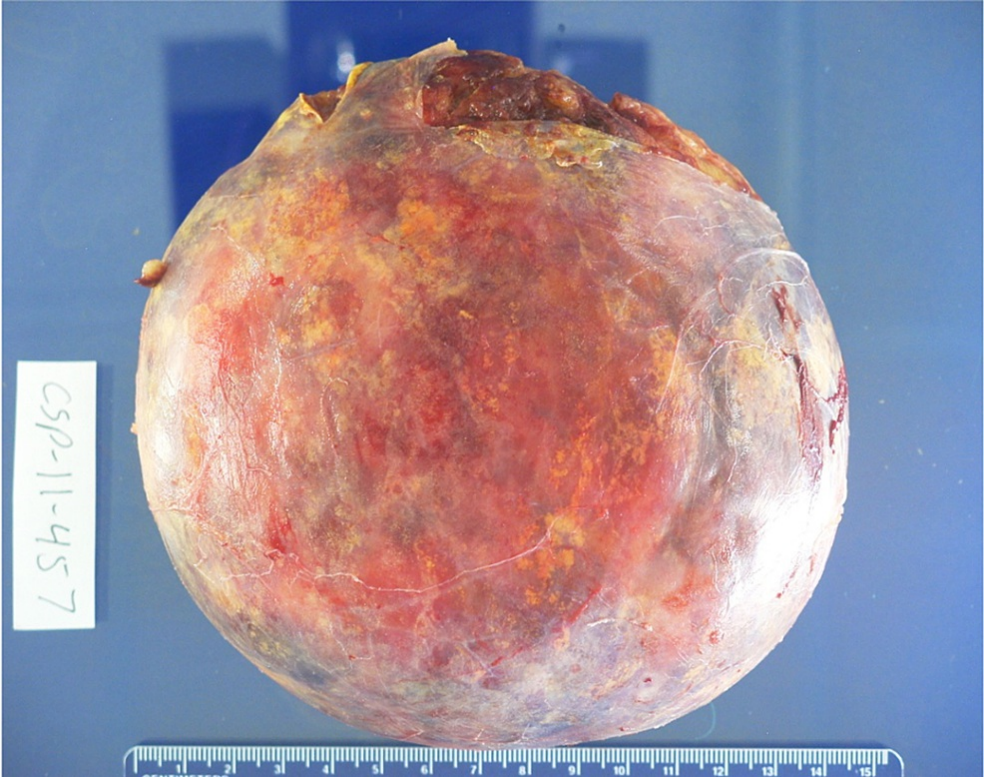
Gross Image of Well Encapsulated Yellow-Tan Adrenal Gland with Myelolipoma The myelolipoma measures 760g, 15.2 x 14.0 cm.

**Figure 9:**
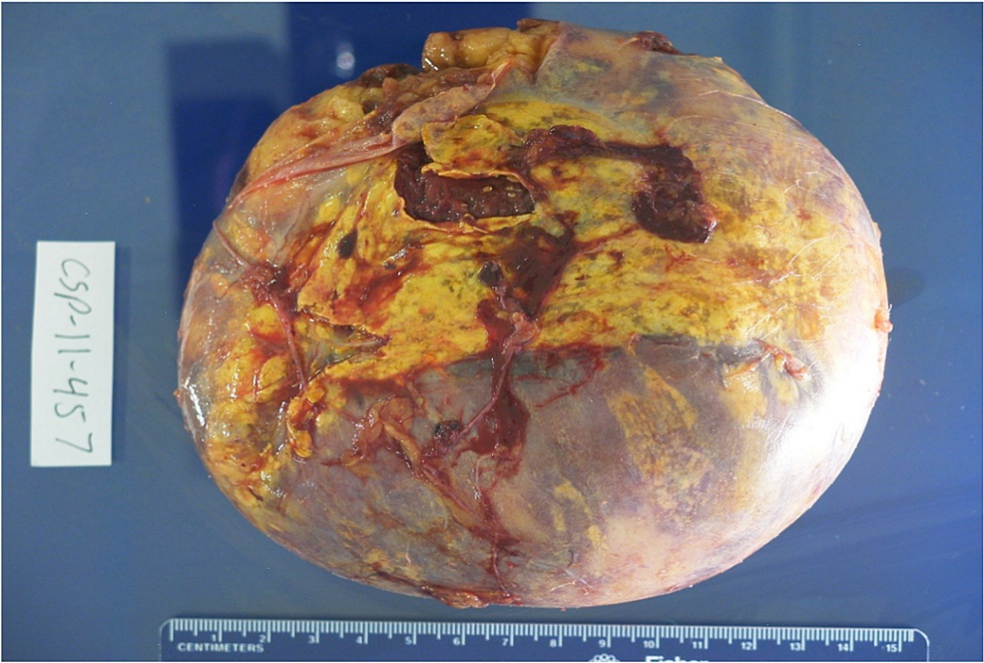
Gross Image Depicting Myelolipoma with Fresh Hemorrhage, Old Hemorrhage, and Fibrosis

**Figure 10:**
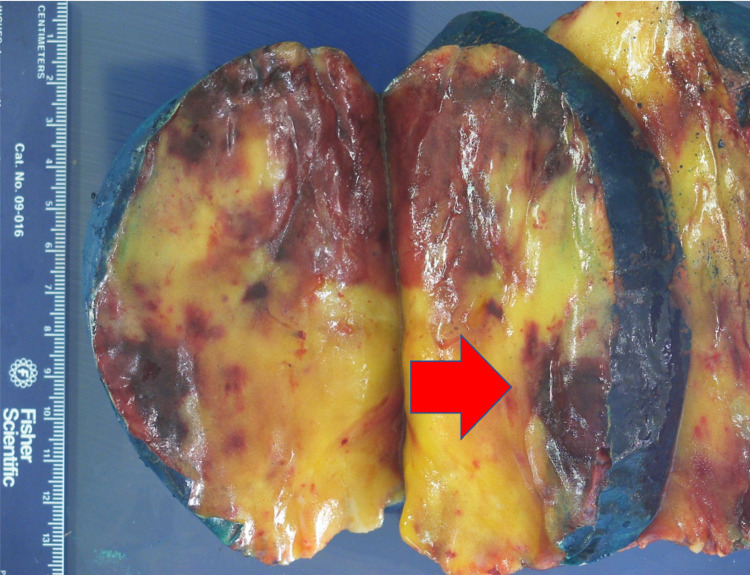
Gross Sectioned Myelolipoma Image demonstrates areas of hemorrhage (red arrow) correlating with the CT and MRI images.

**Figure 11:**
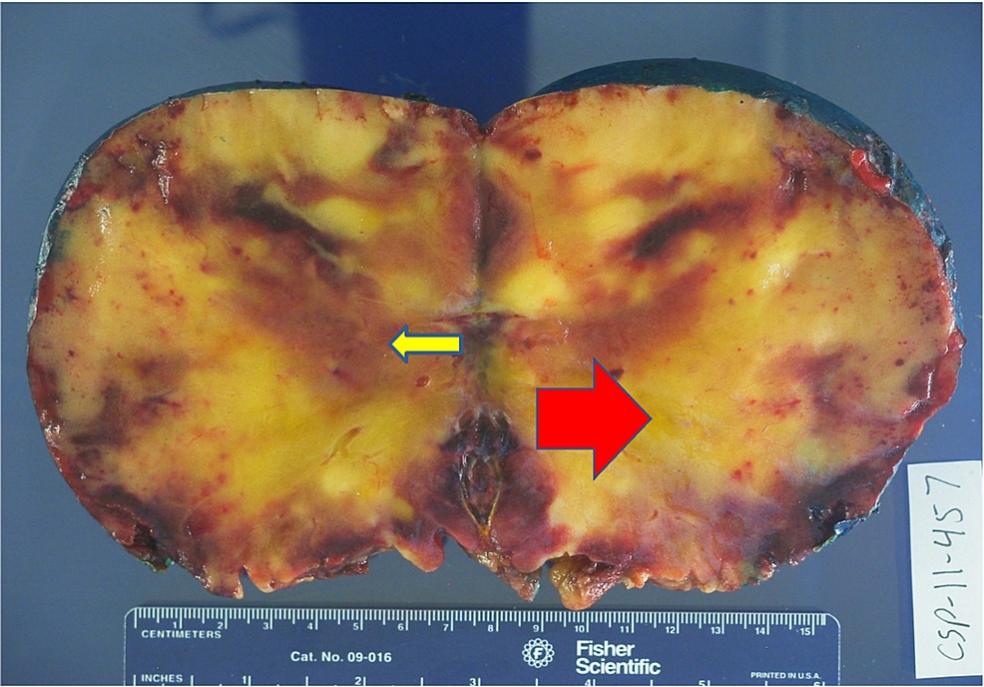
Gross Sectioned Myelolipoma Image demonstrates dark maroon regions of hemosiderin deposition with fatty tissue (red arrow) and myeloid (yellow arrow) elements.

**Figure 12:**
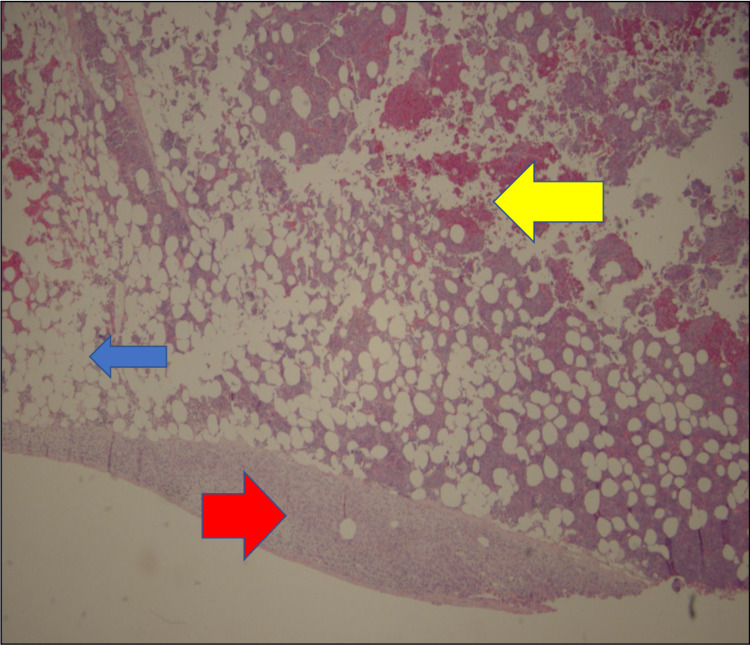
Histology Slide of the Biopsied Myelolipoma Image shows a band of normal adrenal tissue (red arrow) with interspersed myeloid (erythroid/megakaryocytic/lymphoid tissue - yellow arrow), and fat cells (blue arrow).

**Figure 13:**
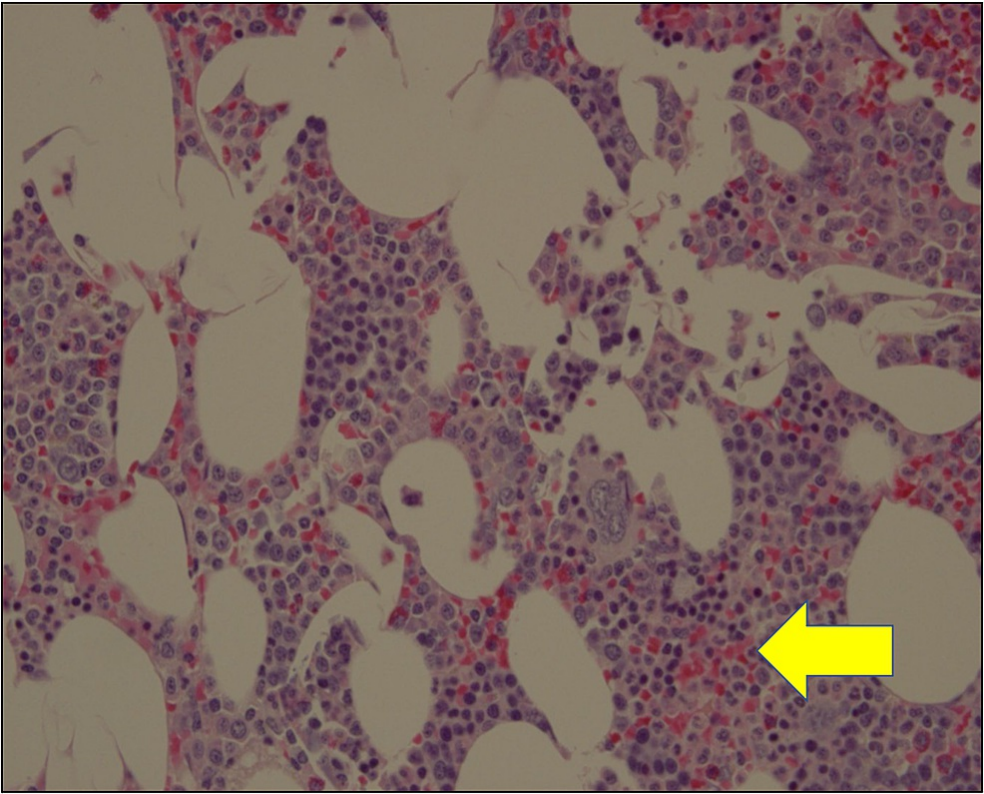
High Power Magnification Myelolipoma Histology Slide Image shows large adipocytes surrounded by clusters of myeloid cells (yellow arrow).

A 50-year-old male with a history of hypertension and diabetes presented with complaints of right flank pain that had been growing increasingly worse over the prior two-week period. The patient was found to be diaphoretic and tachycardic in the emergency room. No other significant findings were noted on physical exam. Emergent multiphasic CT demonstrated a large fatty suprarenal ovoid mass measuring at least 15 cm with areas of high attenuation (Figures 1-3). The mass appeared to arise from the right adrenal fossa displacing the right kidney inferiorly. Subsequent MRI of the abdomen with and without gadolinium confirmed a large, predominantly fat-containing, right adrenal mass with marrow elements and areas of hemorrhage (Figures 4-7). The patient underwent surgical resection, and the post-operative pathology revealed a 760 g well-encapsulated giant hemorrhagic adrenal myelolipoma with fibrosis, and areas of old and fresh hemorrhage (Figures 8-13). The patient is currently disease free and undergoing routine follow-up.

## Discussion

Adrenal myelolipomas are considered a rare finding in radiological imaging, composed of mature lipomatous and hematopoietic tissue. Most of these tumors are < 4 cm when first discovered and many either do not grow or demonstrate slow growth [7]. One study looking at 320 myelolipomas found that the median tumor size minimally increased from 2.3 to 2.6 cm and only 16% of myelolipomas grew more than 1 cm over four and a half years of follow-up [4]. The pathogenesis of these tumors remains unclear. The most widely accepted theory postulates that they arise from metaplasia of adrenal cortical cells when subjected to chronic stress or degeneration [6]. Feng et al. hypothesized that the fat component is derived from mesenchymal stem cells of stromal fat from the adrenal cortex, then circulating hematopoietic cells are recruited possibly due to the release of the granulocyte colony stimulating factor by adrenal cortical tissue [8]. Another study found that the majority of myelolipomas had a non-random X-chromosome inactivation suggesting clonal origin of these tumors [9]. Myelolipomas are generally hormonally silent and clinically asymptomatic [1]. There has been association with endocrine dysfunctions, particularly congenital adrenal hyperplasia (CAH) and adrenocorticotropic hormone (ACTH) secretion [7]. However, endocrine findings are so rare that most patients do not receive evaluation for endocrine dysfunctions [7]. Pain is usually felt if the tumor is larger than 10 cm, due to compression of surrounding structures, or if it presents with hemorrhagic rupture [1]. 

Spontaneous myelolipoma rupture is rare, being reported in 4.5% of myelolipomas [2]. Most ruptures commonly occur in large tumors measuring 10-12 cm with the smallest recorded rupture occurring in a 7-cm tumor [5]. Spontaneous ruptures can be intra-tumoral or extra-tumoral and present with chronic retroperitoneal bleeds or with acute hemorrhagic shock [2]. Intra-tumoral ruptures are more common than extra-tumoral ruptures, which is what occurred in the myelolipoma presented in this case [10]. Underlying factors that may contribute to myelolipoma hemorrhage include diseases that can damage vessels (hypertension, diabetes mellitus, hyperlipidemia, etc.), diseases that decrease procoagulant factors (i.e. cirrhosis), or chronic kidney disease [11].

In this case, a 15-cm tumor was measured with contrast-enhanced CT. US, CT, and MRI are all effective in helping diagnose adrenal myelolipomas, with CT being the most sensitive [12]. Due to their lipid content, myelolipomas present as well-marginated heterogenous masses with attenuation usually less than 0 HU [1]. The attenuation of these tumors are mildly higher than that of ambient fat spaces due to hematopoietic tissue, which enhances on CT (Figure 1) [1]. Acute and subacute hemorrhage presents with high density on non-contrast-enhanced CT with attenuation usually ranging in the 50-90 HU range [1]. Chronic hematomas will appear as a mass with a hypoattenuating center on CT [13]. Myelolipomas contain macroscopic fat which distinguishes them from other adrenal cortical adenomas containing intracellular fat. On MRI macroscopic fat will present with high signal intensity on T1 and intermediate to high signal intensity on T2 [14]. Macroscopic fat will lose signal with the application of fat suppression while signal will usually persist with myeloid elements and hemorrhage contained within the tumor [14]. This distinguishes myelolipomas from adrenal adenomas (most common adrenal lesions) in that adrenal adenomas demonstrate a loss of signal intensity on out-of-phase MRI images compared to in-phase images [15]. This is due to adrenal adenomas containing microscopic fat which is fat within tumor cells as opposed to intra-humoral adipocytes found in macroscopic fat [15]. The onset of hemorrhage can be determined on MRI, with early hemorrhage (< seven days onset) showing up iso-tense on T1 and low signal on T2, subacute hemorrhage (seven days-seven weeks onset) showing high signal on T1 and T2, and late hemorrhage (past seven weeks onset) appearing hypo-intense on both [1]. In this case the diagnosis was confirmed with MRI. T1 fat saturated MRI showed (Figure 4) increased signal of the hemorrhage with loss of signal from the tumor, while T2 non-fat saturated MRI showed low signal intensity of the hemorrhage (Figures 5, 7), indicating that the hemorrhage was relatively acute. Tissue sampling is rarely needed for a definitive diagnosis, however if imaging findings are inclusive, CT-guided biopsy may be performed [14].

No malignant potential, regardless of size, has been reported in a myelolipoma so surgical intervention is not required for asymptomatic and non-hemorrhagic myelolipomas. Conservative management with follow-up over one to two years with imaging to ensure size stability has been recommended. Patients with myelolipomas > 10 cm, and in some studies > 7 cm, are strongly encouraged to undergo surgery due to the increased risk of hemorrhage [14]. In these cases a laparoscopic approach is more superior to the open method as it can lead to lower morbidity and faster recovery and discharge [12]. If acute bleeding occurs, some reports have shown that trans-catheter arterial embolization can stop the bleeding and delay the definitive tumor removal to an elective procedure [2]. Lesions typically do not reoccur following surgical excision [6]. Due to the large right adrenal mass measuring 15 cm on CT, surgery was recommended for this case and follow-up showed the patient to be disease free. Gross pathology reports post-surgery will show capsular/pseudo-capsular yellow (adipose tissue) and red/brown (hematopoietic) cut surfaces with or without hemorrhage (Figures 10, 11) [2]. Histology will show a tumor consisting of mature adipocytes and bone marrow hematopoietic cells with or without internal hemorrhage (Figures 12, 13) [1]. In this case gross pathology and histology were consistent with a hemorrhagic myelolipoma. 

## Conclusions

A giant hemorrhagic adrenal myelolipoma is a rare entity. Hemorrhagic myelolipomas are mostly large and often cause symptomatic flank pain. Imaging modalities such as CT and MRI can yield an accurate diagnosis. With the correct diagnosis the proper treatment can be administered based on the size of the mass and the extent that it bleeds. We submit one more case to the world literature demonstrating excellent radiographic and pathologic correlation.
